# A Study on COMP and CTX-II as Molecular Markers for the Diagnosis of Intervertebral Disc Degeneration

**DOI:** 10.1155/2021/3371091

**Published:** 2021-08-03

**Authors:** Dong-Duo Qi, Zhong-Han Liu, De-Sheng Wu, Yu-Feng Huang

**Affiliations:** Department of Spine Surgery, Shanghai East Hospital, School of Medicine, Tongji University, Shanghai 200092, China

## Abstract

**Background:**

Diagnosis of intervertebral disc degeneration (IVDD) is challenging at the early stage. The cartilage oligomeric matrix protein (COMP) and extracellular matrix degradation products of C-telopeptide of type II collagen (CTX-II) serve as markers for the serological diagnosis of IVDD. Oxidative stress might cause IVDD and matrix degeneration.

**Methods:**

A total of 128 male adult Sprague–Dawley (SD) rats were randomly and equally assigned to the experimental and control groups. The experimental group was used to construct IVDD models by acupuncture, while the control group underwent sham operation. The animals were executed every week for 8 weeks after intervertebral disc acupuncture, and serum samples were collected for the estimation of CTX-II and COMP concentrations by enzyme-linked immunosorbent assay (ELISA). Also, the histological changes and caudal magnetic resonance imaging (MRI) changes were examined in the intervertebral disc.

**Results:**

IVDD in rats worsened with prolonged follow-up after acupuncture. At all the time points, the experimental group showed altered histological and caudal vertebra MRI signals, and serum CTX-II and COMP concentrations were significantly greater than those of the control group. These levels increase with the process of IVDD.

**Conclusion:**

Serum CTX-II and COMP estimation is a reliable method to diagnose IVDD, and their concentrations show a positive correlation with the process of IVDD.

## 1. Background

Neck and lower back pain is the most common reason for patients to seek medical advice. The pain in about 39% of the patients is the result of intervertebral disc disorders [1]. Neck and lower back pain significantly lowers the work efficiency and prolongs the working hours [2], posing heavy medical burdens to the patients. Statistically, the lifetime prevalence of neck and lower back pain is 84%, resulting in disability in 11-12% of patients. The impact of this disability on the medical industry is increasing, with a sharp increase in treatment costs every year. The medical expense on neck and lower back pain exceeds USD 200 billion every year [3, 4].

The treatment strategy for intervertebral disc degeneration- (IVDD-) induced protrusion of the intervertebral disc focuses on alleviating clinical symptoms. Spinal fusion is the gold standard for the treatment of protrusion of the intervertebral disc but causes degeneration at adjacent segments and requires repeated surgeries in some patients. The recent advances in biological therapies, including gene therapy, stem cell-based tissue engineering, IVDD-regulating biological factors, and microRNA therapy, prevent IVDD by promoting extracellular matrix repair and regeneration. With continuous breakthroughs in studies, these new therapies could replace surgery as an early intervention of IVDD but impose high requirements for the early diagnosis of IVDD.

The current diagnosis of protrusion of the intervertebral disc at the neck and lower back is based on the consistent findings in medical history, clinical physical examination, and imaging examination; magnetic resonance imaging (MRI) is an optimal noninvasive imaging method for a definite diagnosis [2, 5]. Furthermore, in clinical practice, IVDD is graded according to different intervertebral disc signals and heights in MRI findings but does not reflect the staging of IVDD [6], and no diagnosis could be made at the early stage of the pathological changes because histopathological changes precede the imaging modifications. Further studies on IVDD showed that IVDD patients had increased expression of extracellular matrix degradation products and some inflammatory mediators in serum or urine. This increase was observed concurrently or before the pathological changes of IVDD, which might serve as the potential molecular markers than MRI findings in the early diagnosis of IVDD. With the development of diagnostic technology, the detection of molecular markers in blood or urine enables the prevention, prognosis, and precise (grading and staging) diagnosis of IVDD.

IVDD is closely related to intervertebral disc senescence. Oxidative stress is the main factor that causes cell senescence. ECM (extracellular matrix) destruction results in the loss of normal biomechanical correlation between intervertebral discs and accelerates disc degeneration. The levels of ECM, type II collagen, and proteoglycan are critical for normal disc function, especially in the nucleus pulposus [7].

C-telopeptide of type II collagen (CTX-II) is one of the leading products of type II collagen degradation during IVDD and is the degradation product of C-telopeptide, the 1/4 segment of type II collagen. The cartilage oligomeric matrix protein (COMP) is a vital member of the platelet cadherin family discovered recently. It consists of five independent subunits and is a pentameric protein linked by disulfide bonds. Prof. Dick Heinegård [8] from Lund University, Sweden, first discovered and reported COMP in the cartilage tissues in 1992. COMP binds to type I collagen, type II collagen, type IX collagen, fibromuscular fibroin, and proteoglycan and plays a key role in the composition, stability, and water molecule transport of cartilage extracellular matrix. Some studies [9, 10] have shown that CTX-II and COMP are potential molecular markers for joint degenerative diseases as detected by serology; however, the correlation between CTX-II and COMP concentrations and IVDD is yet unknown. To evaluate the value of serum COMP and CTX-II as molecular markers for IVDD diagnosis, a longitudinal analysis of the proteins in adult male rats during IVDD was carried out, and the correlation between the concentrations and histological and MRI changes in IVDD was analyzed.

In addition, the concentration of MDA (malondialdehyde) and SOD (superoxide dismutase), oxidative stress markers, was positively correlated with CTX-II and COMP. The level of MDA was correlated positively with that of CTX-II [11] and COMP [12], while the level of SOD [13] was negatively correlated with that of CTX-II and COMP.

## 2. Methods and Materials

### 2.1. Animals

A total of 128 healthy adult Sprague–Dawley (SD) male rats (200 ± 20 g, 2–3 weeks old) were obtained from the Animal Experiment Center of Tongji University (Shanghai, China), because they have thick tails that are convenient for experimental operations and reducing operational error. Aged rats were excluded because they already have spontaneous IVDD. None of the rats were administered any drugs before the experiment. The rats were given at least one week to acclimatize to the surrounding environment. The rats were randomly grouped into the control and experimental groups (*n* = 64/group). The control group was further divided equally into 8 subgroups and fed normally. The experimental group was also randomly and equally grouped into 8 subgroups and subjected to caudal intervertebral disc acupuncture for modeling after one week of adaptive feeding. Following acupuncture surgery, both groups were free to move in the cages; their health status was observed twice every day. This study was approved by the Animal Ethics Committee of Shanghai East Hospital of Tongji University.

### 2.2. Caudal Vertebra Acupuncture

**Figure 1:**
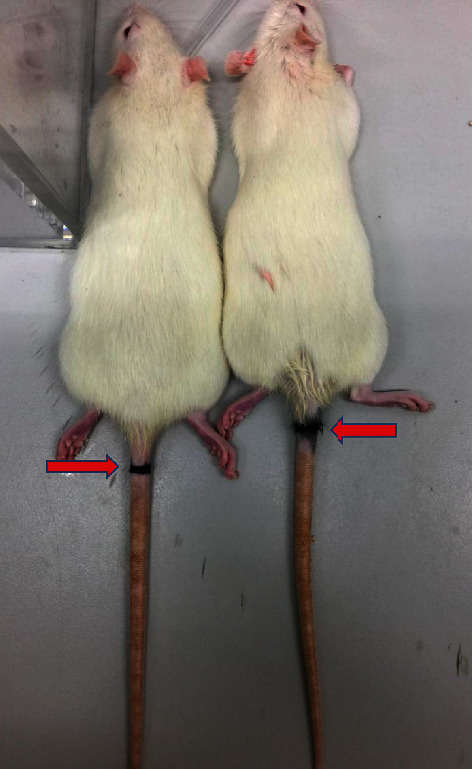
The model of caudal vertebrae was completed by acupuncture. Caudal vertebra acupuncture: the experimental group (2–3 weeks old) rats were anesthetized by inhaled anesthesia: induced anesthesia with 3-4% isoflurane and maintained anesthesia with 1-2% isoflurane (flow rate 0.6-0.8 L/h). When the anesthesia took effect, relevant sites were located anatomically, and the caudal vertebra Co7/8 segment of the intervertebral disc was marked in the rats for the experiment. The arrow indicated the segment for the acupuncture. All male rats were selected to avoid the effect of estrogen on intervertebral disc degeneration (*n* = 8 in each group).

The experimental group (2–3 weeks old) rats were anesthetized by inhaled anesthesia: induced anesthesia with 3-4% isoflurane and maintained anesthesia with 1-2% isoflurane (flow rate 0.6-0.8 L/h). When the anesthesia took effect, relevant sites were located anatomically by X-ray, and the seventh and eighth in caudal vertebra (Co7/8) segment of the intervertebral disc were marked in the rats for subsequent experiments (Figure 1). In the caudal vertebra acupuncture operation, the marked site was disinfected, the SD rat was placed in the prone position, and the intervertebral disc was punctured with a no. 26 needle until the needle reached the contralateral annulus fibrosus. Then, the internal structure of the intervertebral disc was broken by rotating the annulus fibrosus 360° clockwise, and the needle was left in the intervertebral disc for 30 min. Subsequently, the needle was taken out, the skin was disinfected, and the rat was placed in the awakening room, as described previously [14]. To evaluate the disc degeneration, one rat was randomly selected from each of the eight experimental groups postoperatively. These rats were euthanized for further histological evaluation.

### 2.3. Hematoxylin-Eosin (H&E) Staining and Safranin O-Fast Green FCF Staining

A total of 8 rats from the experimental and control groups were sacrificed, respectively, every week for 8 weeks after modeling by intervertebral disc acupuncture. The caudal vertebra was cut open in each rat; the intervertebral disc layer was removed, fixed in 10% buffer formalin for 72 h, decalcified by EDTA (ethylenediaminetetraacetic acid) for 4 weeks, and stained with HE and safranin O-fast green FCF. One pathologist not aware of the grouping was engaged to evaluate the histopathological changes of the intervertebral disc, as described previously [14].

### 2.4. CTX-II and COMP Concentration Measurement

A total of eight rats with fasting overnight from the experimental and control groups were sacrificed, respectively, every week for 8 weeks after modeling by intervertebral disc acupuncture. A blood sample of 5 mL was withdrawn from the eyeball of each rat in the control or experimental group, and the serum was collected by centrifugation at 2000 × *g* at room temperature for 15 min and stored at -80°C until further use (CTX-II and COMP did not depend on the measurement time). The serum COMP and CTX-II levels were estimated by enzyme-linked immunosorbent assay (ELISA; rat serum COMP detection kit and rat serum CTX-II detection kit, Cosmo Bio, Carlsbad, CA, USA).

### 2.5. MRI Analysis

Eight animals from each group were sacrificed, respectively, at every week for 8 weeks. All the animals were subjected to caudal vertebra MRI on a Philips 1.5T superconducting MRI scanner to elucidate the morphological changes of the vertebral and coccygeal tissues and the signal changes in the intervertebral disc reflected in MRI. The MRI findings were read randomly and presented by different imaging physicians in a single-blind manner and analyzed using the computer image reading analysis system (IMPAX 6.5.3.3009 Radiologist with QC). The high signal areas of the entire intervertebral disc were extracted. The images were selected randomly, and each selected image was measured three times.

### 2.6. Immunohistochemical Staining

A total of 8 rats in the experimental and control groups were sacrificed, respectively, every week for 8 weeks after modeling by intervertebral disc acupuncture. The caudal vertebra was cut open in each rat, and the intervertebral disc layer was removed, fixed in 10% buffer formalin for 72 h, and decalcified by EDTA for 4 weeks. The sections were deparaffinized and rehydrated, and then, antigen was retrieved in the microwave for 15 min each. Then, endogenous peroxidase activity was blocked for 10 min by 3% hydrogen peroxide, and nonspecific binding sites were blocked for 30 min at room temperature by 5% BSA. Then, the sections were incubated with primary antibodies: MMP3 (ab268084, Abcam, Cambridge, UK), aggrecan (ab216965, Abcam, Cambridge, UK), and Col2a1 (ab188570, Abcam, Cambridge, UK) at 4°C overnight, followed by secondary antibodies. Finally, the slides were sealed and photographed under a microscope.

### 2.7. Statistical Analysis

Prism 5.0 (GraphPad Software Inc., San Diego, CA, USA) and SPSS 20.0 (SPSS Inc., Chicago, IL, USA) software were used for statistical analysis. The measurement data were expressed as mean ± standard deviation, and the enumeration data were evaluated by nonparametric statistics. Chi-square and exact probability tests were used to perform row-column (*R* × *C*) cross-tabulation. *P* < 0.05 indicated a statistical significance.

## 3. Results

### 3.1. MRI Findings Showed Increasingly Significant Degeneration of the Acupunctured Segment in Rats over Time

**Figure 2:**
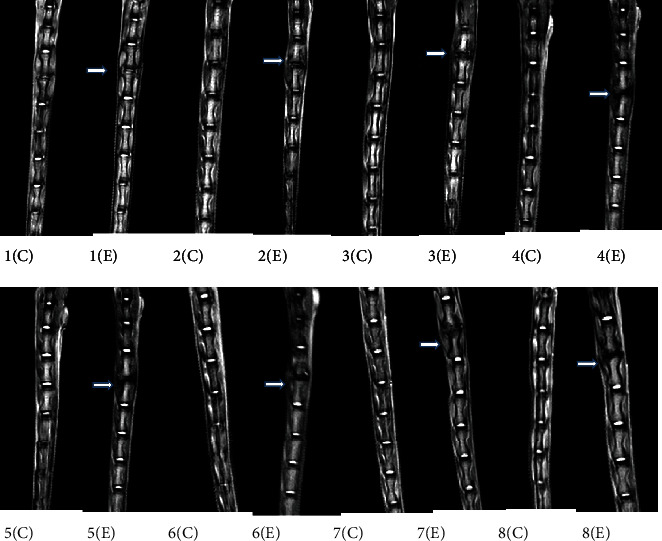
MRI findings showed increasingly significant degeneration of the acupunctured segment in rats over time (*n* = 8 in each group). 1(C) and 1(E): 1-week flowing acupuncture in the control and experimental groups. 2(C) and 2(E): 2-week flowing acupuncture in the control and experimental groups. 3(C) and 3(E): 3-week flowing acupuncture in the control and experimental groups. 4(C) and 4(E): 4-week flowing acupuncture in the control and experimental groups. 5(C) and 5(E): 5-week flowing acupuncture in the control and experimental groups. 6(C) and 6(E): 6-week flowing acupuncture in the control and experimental groups. 7(C) and 7(E): 7-week flowing acupuncture in the control and experimental groups. 8(C) and 8(E): 8-week flowing acupuncture in the control and experimental groups. The rats were sacrificed in each week until week 8, and all the injured segments were examined by MRI. The MRI findings showed that the severity of IVDD (the nucleus pulposus (NPC) structure changed from a uniform and bright white to uneven and black, the boundary between NPC and annulus fiber disappeared, the MRI T2 phase changed from high signal to low signal, and the height of the intervertebral disc decreased progressively) increased over time from week 1 to week 8.

After modeling by acupuncture at the same site in the caudal vertebra 7-8 (Co7-8), the rats were sacrificed every week for 8 weeks and examined by MRI scan. The findings showed that the degeneration of the acupunctured segment in the rat worsened over time; the severity of IVDD increased; the nucleus pulposus (NP) structure changed from a uniform and bright white to uneven and black; the boundary between NPC and annulus fiber disappeared; the MRI T2 phase changed from high signal to low signal, and the height of the intervertebral disc decreased progressively (Figure 2).

### 3.2. H&E Staining and Safranin O-Fast Green Staining Showed Severe IVDD in the Rats of the Experimental Group

**Figure 3:**
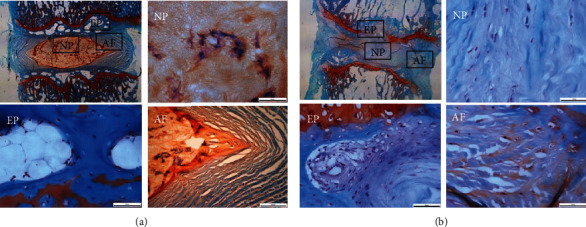
Safranin O-fast green staining showed severe IVDD in the rats of the experimental group. (a) The control group showed complete NPCs in the intervertebral disc, a quasicircular central NPC, a large number of notochord cells, a small number of chondroid cells, and the peripheral annulus fibrosus arranged in an annular manner with a clear boundary with the NPCs. (b) The experimental group (acupunctured group) showed the annulus fibrosus disrupted and disorganized over time (the inner fiber of the annulus fibrosus twisted and protruded into the intervertebral disc with an unclear boundary with the NPCs and the cracks inside NPCs) and the chondroid cells were replaced by fibrocartilage cells and inflammatory cell infiltration. AF: annulus fibrosus; NP: nucleus pulposus; EP: endplate cartilage.

**Figure 4:**
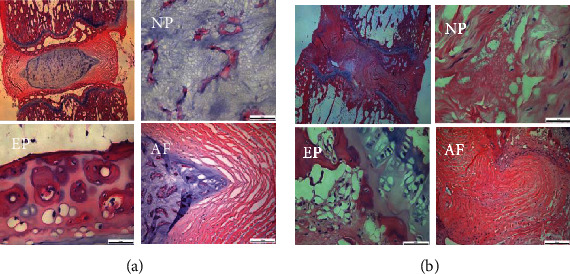
H&E staining showed severe IVDD in the rats of the experimental group. (a) The control group showed no significant difference was detected between different time points. (b) The experimental group showed the annulus fibrosus was manifested by annular disruption, NPC loss, endplate calcification, and cell necrosis. AF: annulus fibrosus; NP: nucleus pulposus; EP: endplate cartilage. *n* = 8 in each group.

According to safranin O-fast green staining results, no significant difference was detected among different time points in the control group. Moreover, the control group showed intact NPCs in the intervertebral disc: a quasicircular central NPC, a large number of notochord cells, a small number of chondroid cells, and the peripheral annulus fibrosus arranged in an annular manner with a distinct boundary with the NPCs. Conversely, the experimental group showed varied degeneration in the NPCs of the intervertebral disc: the annulus fibrosus worsened over time, the inner fiber of the annulus fibrosus twisted and protruded into the intervertebral disc with an unclear boundary with the NPCs and the cracks inside NPCs, and the chondroid cells were replaced by fibrocartilage cell and inflammatory cell infiltration. Consistently, examination by H&E staining revealed that the experimental group showed degeneration of the NPCs, and the annulus fibrosus was manifested by annular disruption, NPC herniation, endplate calcification and thickening, hyaline membrane thinning, and cartilage cell necrosis (Figures 3 and 4).

### 3.3. Expressions of Aggrecan and Type II Collagen Decreased, and the Expression of Matrix Metalloproteinase-3 (MMP3) Increased with the Progress of IVDD

**Figure 5:**
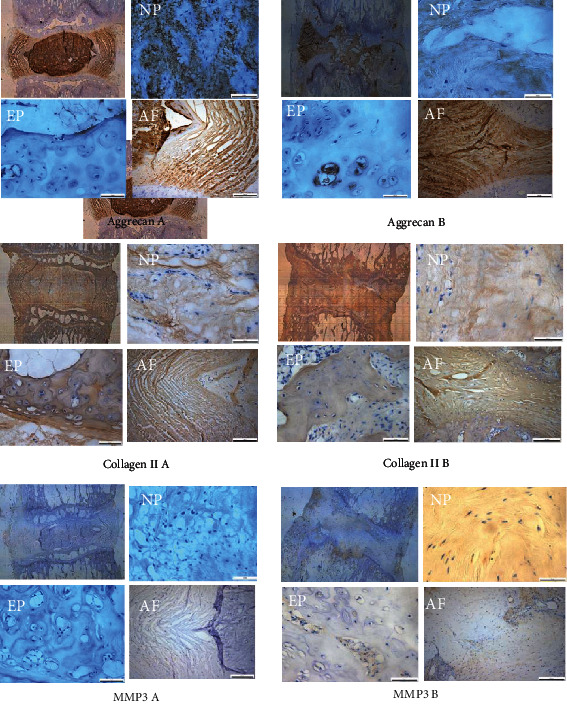
The expressions level of aggrecan and type II collagen decreased, and the expression of MMP3 increased with the progress of IVDD. Immunohistochemistry showed that the expression levels of aggrecan and type II collagen are significantly decreased. Immunohistochemistry showed that the level of MMP3 in the caudal intervertebral disc of (b) experimental rats is significantly increased than that of the (a) control group.*n* = 8 in each group.

Immunohistochemical staining results of aggrecan and type II collagen in the caudal intervertebral disc of rats showed that NPC tissues were absent or partially missing in the degenerative rats, and the annulus fibrosus and endplate structure were damaged. Aggrecan and type II collagen were clearly expressed in the cytoplasm of the cartilage endplate, NPC, and annulus fibrosus, and the expression levels were higher in the normal intervertebral disc than those in the degenerative groups. The immunohistochemical staining results of MMP3 in the caudal intervertebral disc of rats showed that NPC tissues were absent or partially missing in the degenerative rats and the annulus fibrosus and the endplate structure were damaged. The expression of MMP3 was mainly expressed in the cytoplasm, and the experimental group had a significantly higher MMP3 expression than the control group. These findings may indicate that type II collagen in the NPC and annulus fibrosus was degraded and gradually replaced by type I collagen with the progress of degeneration. Moreover, the increased expression of matrix metalloproteinase (MMP) in the degenerated group suggested the degenerated pathology during IVDD (Figure 5).

### 3.4. Serum CTX-II Concentration Increased with the Process of IVDD

**Figure 6:**
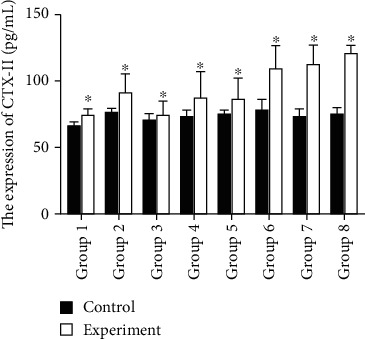
Serum CTX-II concentration increased with the process of IVDD. Group 1: 1-week flowing acupuncture in the control and experimental groups. Group 2: 2-week flowing acupuncture in the control and experimental groups. Group 3: 3-week flowing acupuncture in the control and experimental groups. Group 4: 4-week flowing acupuncture in the control and experimental groups. Group 5: 5-week flowing acupuncture in the control and experimental groups. Group 6: 6-week flowing acupuncture in the control and experimental groups. Group 7: 7-week flowing acupuncture in the control and experimental groups. Group 8: 8-week flowing acupuncture in the control and experimental groups. The level of CTX-II concentration is significantly increased with time following injury. The concentration of CTX-II in groups 1-8 is listed in Table 1. A statistically significant difference was detected in the increase in CTX-II concentration between the experimental and control groups (*P* < 0.05) (*n* = 8 in each group).

The experimental group showed an increase in CTX-II concentration (pg/mL), which was 73.95 ± 5.53, 90.71 ± 13.5, 74.48 ± 10.38, 87.35 ± 20.256, 86.16 ± 15.95, 108.59 ± 17.45, 111.27 ± 15.08, and 119.16 ± 6.95 at weeks 1–8, respectively. A statistically significant difference was detected in the increase in CTX-II concentration between the experimental and control groups (*P* < 0.05, *n* = 128) (Figure 6, Table 1).

### 3.5. Serum COMP Concentration Increased with the Development of IVDD

**Figure 7:**
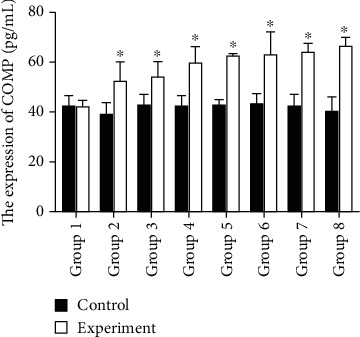
Serum COMP concentration increased with the development of IVDD. Group 1: 1-week flowing acupuncture in the control and experimental groups. Group 2: 2-week flowing acupuncture in the control and experimental groups. Group 3: 3-week flowing acupuncture in the control and experimental groups. Group 4: 4-week flowing acupuncture in the control and experimental groups. Group 5: 5-week flowing acupuncture in the control and experimental groups. Group 6: 6-week flowing acupuncture in the control and experimental groups. Group 7: 7-week flowing acupuncture in the control and experimental groups. Group 8: 8-week flowing acupuncture in the control and experimental groups. The level of COMP concentration is significantly increased with time following injury. The concentration of CTX-II in groups 1-8 is listed in Table 2. A statistically significant difference was detected in the increase in COMP concentration between the experimental and control groups (*P* < 0.05) (*n* = 8 in each group).

The experimental group showed an increase in the COMP concentration (pg/mL), which was 41.71 ± 2.86, 51.94 ± 7.64, 53.37 ± 6.3, 59.37 ± 6.3, 61.94 ± 1.23, 62.63 ± 8.24, 63.71 ± 3.52, and 65.95 ± 2.86 at weeks 1–8, respectively. A statistically significant difference was detected in the increase in COMP concentration between the experimental and control groups (*P* < 0.05, *n* = 128) (Figure 7, Table 2).

### 3.6. Serum MDA Concentration Increased and SOD Concentration Decreased with the Development of IVDD

**Figure 8:**
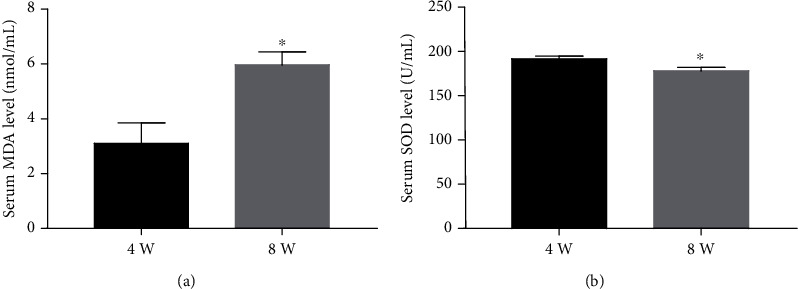
The serum concentration of MDA and SOD is increased with the extent of IVDD. (a) The serum MDA level in the control and experimental groups at 4 weeks and 8 weeks following acupuncture. (b) The serum SOD level in the control and experimental groups at 4 weeks and 8 weeks following acupuncture (*n* = 3, ^∗^*P* < 0.05).

Reactive oxygen species (ROS) production is inevitable due to the oxygen-utilizing metabolism of disc cells despite the hypoxic microenvironment in IVDD. Reportedly, CTX-II and COMP are closely related to oxidative stress, which was further verified in IVDD based on the correlation study between MDA and SOD and IVDD. We found a correlation between the level of MDA and SOD and oxidative stress markers with the development of IVDD. The serum MDA concentration at 8 w after modeling was significantly higher than that at 4 w (*n* = 3, ^∗^*P* < 0.05) (Figure 8(a)), while the serum SOD concentration at 8 w after modeling was significantly lower than that at 4 w (*n* = 3, ^∗^*P* < 0.05) (Figure 8(b)).

## 4. Discussion

The spine is one of the load-bearing structures in the human body, and the intervertebral disc is vital in maintaining mechanical transmission and spinal movement in humans. In this process, the extracellular matrix in the intervertebral disc maintains the smoothness of the intervertebral disc cartilage structure and buffering forces. The intervertebral disc is composed of the endplate cartilage, NPC, and annulus fibrosus. The extracellular matrix in the intervertebral disc is mainly composed of proteoglycan and type II and type I collagen, with proteoglycan as one of the critical components.

The serum CTX-II and COMP concentrations increase in the experimental group with the development of IVDD. Subsequently, the degeneration of the acupunctured segment in the rat worsened, and CTX-II and COMP concentrations were positively correlated with IVDD. Type II collagen is a triple-helix protein composed of three identical polypeptide chain-*α* chains and a significant macromolecule in the extracellular matrix in the intervertebral disc. The initial form of the *α*-chain is the *α*-chain precursor composed of the propeptides at the amino terminal and carboxy terminal, the triple helix in the middle, and the telopeptide between the propeptides and the triple-helix structure [15]. During modification, the propeptides at both terminals are hydrolyzed by enzymes, leaving only the triple-helix structure and the telopeptide, which turn into collagen precursors that crosslinks to form the collagen fiber network.

Type II collagen plays a critical role in maintaining structural stability and functional integrity of the intervertebral disc ^[16]^. The degradation of the extracellular matrix is one of the major pathological changes during IVDD, while the degradation of type II collagen in NPC and the annulus fibrosus and its replacement by type I collagen are the leading pathological manifestations of IVDD [16, 17]. Furthermore, during IVDD, various MMPs, including MMP1, MMP3, and MMP13, occur predominantly during the degradation and change in type II collagen [18]. Under the effect of MMPs, type II collagen is hydrolyzed and forms a type II 3/4 segment of 794-amino acid length and a type II 1/4 fragment of 266 amino acids. Subsequently, the triple-helix structures of these two segments are uncoiled and further degraded by other MMPs. The end products of type II collagen mainly include CTX-II, type II neoepitope (TIINE), and nitrated type II collagen-1 (Coll2-1NO2), among which CTX-II is one of the main products of type II collagen in the intervertebral disc degraded via MMPs and the degradation product of the telopeptide of type II collagen 1/4 segment at the carboxyl terminal [19]. Therefore, with the development of IVDD, the external blood vessels expand continually, releasing serum CTX-II into blood, which serves as the theoretical basis for using serum or urinary molecular markers to diagnose IVDD.

Current studies have shown that serum and urinary CTX-II are potential molecular markers for osteoarthritis and rheumatoid arthritis. Another study showed that the expression of serum CTX-II was significantly increased and positively correlated with MMP3 in rheumatoid arthritis, deeming it as a vital indicator to judge the severity and prognosis of rheumatoid arthritis [20–22]. Furthermore, serum and urinary CTX-II concentrations were positively correlated with the degree of surface cartilage destruction, K-L score, and the degree of joint space narrowing in osteoarthritis, indicating that CTX-II is an optimal molecular marker to judge the prognosis of osteoarthritis [23]. In addition, serum and urinary CTX-II concentrations have been shown to gradually decrease, suggesting that these could be the potential markers for the treatment efficacy on osteoarthritis [24] and rheumatoid arthritis [22, 25], as well as reliable markers for recovery and healing. Similar conclusions have been drawn on serum and urinary CTX-II concentrations in IVDD. Garnero et al. [26] studied the urine of 324 postmenopausal females and found that the urinary CTX-II concentration was between 166 and 299 ng/mmol and closely related to the intervertebral space narrowing, knee osteoarthritis with clinical symptoms, and hand osteoarthritis. A recent study by Brayda-Bruno et al. [27] compared serum vitamin D, CTX-I, and CTX-II concentrations in 79 males with and without IVDD and found that active serum vitamin D and serum CTX-II concentrations were rhythmically distributed over a year. Also, the serum CTX-I concentration was positively correlated with the active serum vitamin D concentration and negatively with serum CTX-II concentration, and the IVDD patients with osteochondrosis showed that serum CTX-II concentration was significantly higher than in those without IVDD and osteochondrosis. Meulenbelt et al. [28] analyzed the urinary CTX-II concentration in 302 patients and found that it was closely correlated with knee osteoarthritis, hip osteoarthritis, and IVDD.

Recent studies have shown that COMP is correlated with articular cartilage destruction and extracellular matrix degradation. Some studies have shown that COMP is a bone marker for knee osteoarthritis, hip osteoarthritis, and rheumatoid arthritis due to its correlation with cartilage destruction and matrix degradation. It can also be a critical molecular marker to judge the prognosis and treatment efficacy on knee and hip osteoarthritis. In the extracellular matrix of the intervertebral disc, COMP binds to type II collagen and proteoglycan to stabilize the cartilage collagen network to maintain the integrity of the intervertebral disc [29]. Taken together, it could be deduced that COMP is a macromolecule released into the blood during cartilage matrix destruction and degradation similar to CTX-II. Goode et al. [30] analyzed the serum COMP, hyaluronic acid, and urinary CTX-II concentrations in 547 patients and found that the serum hyaluronic acid concentration is correlated to the intervertebral space narrowing in females, the urinary CTX-II concentration is correlated with the intervertebral space narrowing, and the serum COMP concentration is closely correlated to the lower back pain induced by intervertebral space narrowing.

Similar to osteoarthritis and rheumatoid arthritis, IVDD is a degenerative disease of the cartilage manifested as extracellular matrix degradation [31]. Therefore, CTX-II and COMP, the molecules released into the blood after extracellular matrix degradation, are potential molecular biomarkers for IVDD. Several studies have focused on CTX-II and COMP in osteoarthritis and rheumatoid arthritis [32], and only a few have reported the specific correlation between CTX-II and COMP. The liability of these molecules as the clinical markers for IVDD needs further exploration in animal and clinical studies.

Oxidative stress is speculated as one of the causes of IVDD. Reactive oxygen species (ROS) production is inevitable due to oxygen-utilizing metabolism of disc cells despite the hypoxic microenvironment in IVDD [33]. IVDD is mainly caused by the changes in nucleus pulposus cells and extracellular matrix. The destruction of ECM leads to the loss of normal biomechanical correlations between intervertebral discs and accelerates the degeneration of intervertebral discs [34]. The degradation of proteoglycan decreases the osmotic pressure of the ECM and the decrease in the NP water content in the nucleus pulposus. In NP, type II collagen is converted to type I collagen [7]. Therefore, it can be concluded that oxidative stress is closely related to the expression levels of CTX-II and COMP during intervertebral disc degenerative changes. Recent studies have shown that oxidative stress promotes the production of apoptotic bodies (Abs) in endplate chondrocytes in rats to increase the expressions of relevant ossification genes, such as *Runx2*, in endplate chondrocytes [35], and the expressions of type X collagen (ColX), Runx2, osteoprotegerin, and alkaline phosphatase (ALP) were significantly higher in degenerative intervertebral discs than in normal intervertebral discs, suggesting that IVDD involves proliferative differentiation similar to that in advanced osteoarthritis [36]. The current study showed that the concentration of MDA and SOD, oxidative stress markers, was correlated to the development of IVDD. The activity of serum and intervertebral disc SOD decreased and MDA increased gradually with disc degeneration [37]. Therefore, the oxidative stress could be a reason for elevated CTX-II and COMP. Watari et al. [11] demonstrated that the serum MDA concentration was correlated to that of CTX-II concentration, but not to CPII concentration and significantly correlated to the serum lipid (total cholesterol and triglyceride) level and body weight. These findings indicated that oxidative stress might be involved in the degradation of type II collagen in the articular cartilage. Coustry et al. [38] utilized MT-COMP pseudoachondroplasia (PSACH) mouse models and found that the accumulation of misfolded COMP in the endoplasmic reticulum of growth plate chondrocytes resulted in premature death and loss of linear growth of chondrocytes. Intriguingly, the premature death of chondrocytes was the result of activated oxidative stress and inflammation via the CHOP-er pathway, which disrupted the adipogenesis-osteogenesis balance. These phenomena suggested that the increase in CTX-II and COMP concentrations might be correlated with enhanced oxidative stress in the intervertebral disc.

Therefore, the detection of serum and urinary molecular markers is a reliable method to diagnose IVDD. However, IVDD is similar to degenerative diseases of the cartilage, such as osteoarthritis, arising from extracellular matrix degradation and inflammatory responses. Thus, serum and urinary molecular markers may not be specific for IVDD diagnosis, thereby necessitating continuous anatomical and physiological analyses of the intervertebral disc in osteoarthritis in several clinical and animal experiments. Substances specific to intervertebral disc tissues may be identified in future studies based on the anatomy and physiology of the disc and emphasize the significance of the diagnosis of intervertebral disc-related diseases according to serum and urine detection results.

## 5. Conclusion

CTX-II and COMP are potential indicators for IVDD in rats, and their expression is a positive correlation with the degeneration extent of IVD.

## Figures and Tables

**Table 1:** The expression level of serum CTX-II in the control group and the experimental group in each week following acupuncture.

Time CTX-II	Control group (pg/mL)	Experimental group (pg/mL)	*P*
1 w	65.59 ± 3.25	73.95 ± 5.53	<0.05
2 w	75.66 ± 3.80	90.71 ± 13.5	<0.05
3 w	70.18 ± 4.35	74.48 ± 10.38	<0.05
4 w	72.94 ± 5.39	87.35 ± 20.256	<0.05
5 w	74.73 ± 4.18	86.16 ± 15.95	<0.05
6 w	77.32 ± 8.47	108.59 ± 17.45	<0.05
7 w	72.4 ± 6.54	111.27 ± 15.08	<0.05
8 w	73.95 ± 5.5	119.16 ± 6.95	<0.05

*N* = 8 in each group. CI: 95% confidence interval.

**Table 2:** The expression level of serum COMP in the control group and the experimental group in each week following acupuncture.

Time COMP	Control group (pg/mL)	Experimental group (pg/mL)	*P*
1 w	41.41 ± 4.39	41.71 ± 2.86	>0.05
2 w	38.22 ± 5.34	51.94 ± 7.64	<0.05
3 w	40.32 ± 4.3	53.37 ± 6.3	<0.05
4 w	41.83 ± 4.5	59.37 ± 6.3	<0.05
5 w	41.85 ± 2.68	61.94 ± 1.23	<0.05
6 w	42.93 ± 4.4	62.63 ± 8.24	<0.05
7 w	41.52 ± 4.82	63.71 ± 3.52	<0.05
8 w	39.52 ± 5.59	65.95 ± 2.86	<0.05

*N* = 8 in each group. CI: 95% confidence interval.

## Data Availability

The data used to support the findings of this study are available from the corresponding authors (Yu-Feng Huang and De-Sheng Wu) upon request.

## References

[1] DePalma M. J., Ketchum J. M., Saullo T. R. (2011). Etiology of chronic low back pain in patients having undergone lumbar fusion. *Pain Medicine*.

[2] Kreiner D. S., Hwang S. W., Easa J. E. (2014). An evidence-based clinical guideline for the diagnosis and treatment of lumbar disc herniation with radiculopathy. *The Spine Journal*.

[3] Jacobs W. C., Rubinstein S. M., Willems P. C. (2013). The evidence on surgical interventions for low back disorders, an overview of systematic reviews. *European Spine Journal*.

[4] Martins D. E., Astur N., Kanas M., Ferretti M., Lenza M., Wajchenberg M. (2016). Quality assessment of systematic reviews for surgical treatment of low back pain: an overview. *The Spine Journal*.

[5] Hamilton-Bennett S. E., Behr S. (2019). Clinical presentation, magnetic resonance imaging features, and outcome in 6 cats with lumbar degenerative intervertebral disc extrusion treated with hemilaminectomy. *Veterinary Surgery*.

[6] Zeckser J., Wolff M., Tucker J., Goodwin J. (2016). Multipotent mesenchymal stem cell treatment for discogenic low back pain and disc degeneration. *Stem Cells International*.

[7] Dowdell J., Erwin M., Choma T., Vaccaro A., Iatridis J., Cho S. K. (2017). Intervertebral disk degeneration and repair. *Neurosurgery*.

[8] Saxne T., Heinegård D. (1992). Cartilage oligomeric matrix protein: a novel marker of cartilage turnover detectable in synovial fluid and blood. *British journal of rheumatology*.

[9] Toda T., Sugioka Y., Koike T. (2020). Soybean isoflavone can protect against osteoarthritis in ovariectomized rats. *Journal of food science and technology*.

[10] Vos L. M., Kuijer R., Huddleston Slater J. J., Stegenga B. (2013). Alteration of cartilage degeneration and inflammation markers in temporomandibular joint osteoarthritis occurs proportionally. *Journal of oral and maxillofacial surgery: official journal of the American Association of Oral and Maxillofacial Surgeons*.

[11] Watari T., Naito K., Sakamoto K., Kurosawa H., Nagaoka I., Kaneko K. (2011). Evaluation of the effect of oxidative stress on articular cartilage in spontaneously osteoarthritic STR/OrtCrlj mice by measuring the biomarkers for oxidative stress and type II collagen degradation/synthesis. *Experimental and Therapeutic Medicine*.

[12] Shafiey S. I., Mohamed W. R., Abo-Saif A. A. (2018). Paroxetine and rivastigmine mitigates adjuvant-induced rheumatoid arthritis in rats: impact on oxidative stress, apoptosis and RANKL/OPG signals. *Life sciences*.

[13] Sun Y., Wang C., Gong C. (2020). Repairing effects of glucosamine sulfate in combination with etoricoxib on articular cartilages of patients with knee osteoarthritis. *Journal of orthopaedic surgery and research.*.

[14] Li X., Yang S., Han L., Mao K., Yang S. (2020). Ciliary IFT80 is essential for intervertebral disc development and maintenance. *The FASEB Journal*.

[15] Willems N., Tellegen A. R., Bergknut N. (2016). Inflammatory profiles in canine intervertebral disc degeneration. *BMC veterinary research*.

[16] Wu Q., Mathers C., Wang E. W., Wenkert D., Huang J. H., Sheng S. (2019). TGF-*β* initiates *β*-catenin-mediated CTGF secretory pathway in old bovine nucleus pulposus cells: a potential mechanism for intervertebral disc degeneration. *JBMR Plus*.

[17] Molinos M., Almeida C. R., Caldeira J., Gonçalves R. M., Barbosa M. A., Cunha C. (2015). Inflammation in intervertebral disc degeneration and regeneration. *Journal of the Royal Society Interface*.

[18] Mayer J. E., Iatridis J. C., Chan D., Gottesman O., Hecht A. C., Qureshi S. A. (2013). Genetic polymorphisms associated with intervertebral disc degeneration. *The Spine Journal*.

[19] Park Y. M., Kim S. J., Lee K. J., Yang S. S., Min B. H., Yoon H. C. (2015). Detection of CTX-II in serum and urine to diagnose osteoarthritis by using a fluoro-microbeads guiding chip. *Biosensors & Bioelectronics*.

[20] Wang J. R., Gao W. N., Grimm R. (2017). A method to identify trace sulfated IgG N-glycans as biomarkers for rheumatoid arthritis. *Nature Communications*.

[21] Vosse D., Landewe R., Garnero P., van der Heijde D., van der Linden S., Geusens P. (2008). Association of markers of bone- and cartilage-degradation with radiological changes at baseline and after 2 years follow-up in patients with ankylosing spondylitis. *Rheumatology (Oxford)*.

[22] Landewé R., Geusens P., Boers M. (2004). Markers for type II collagen breakdown predict the effect of disease-modifying treatment on long-term radiographic progression in patients with rheumatoid arthritis. *Arthritis and Rheumatism*.

[23] Attur M., Krasnokutsky-Samuels S., Samuels J., Abramson S. B. (2013). Prognostic biomarkers in osteoarthritis. *Current Opinion in Rheumatology*.

[24] Wang P., Song J., Qian D. (2019). CTX-II and YKL-40 in early diagnosis and treatment evaluation of osteoarthritis. *Experimental and Therapeutic Medicine*.

[25] Hao H. Q., Zhang J. F., He Q. Q., Wang Z. (2019). Cartilage oligomeric matrix protein, C-terminal cross-linking telopeptide of type II collagen, and matrix metalloproteinase-3 as biomarkers for knee and hip osteoarthritis (OA) diagnosis: a systematic review and meta-analysis. *Osteoarthritis and Cartilage*.

[26] Garnero P., Sornay-Rendu E., Arlot M., Christiansen C., Delmas P. D. (2004). Association between spine disc degeneration and type II collagen degradation in postmenopausal women: the OFELY study. *Arthritis and Rheumatism*.

[27] Brayda-Bruno M., Viganò M., Cauci S. (2017). Plasma vitamin D and osteo-cartilaginous markers in Italian males affected by intervertebral disc degeneration: focus on seasonal and pathological trend of type II collagen degradation. *Clinica Chimica Acta*.

[28] Meulenbelt I., Kloppenburg M., Kroon H. M. (2006). Urinary CTX-II levels are associated with radiographic subtypes of osteoarthritis in hip, knee, hand, and facet joints in subject with familial osteoarthritis at multiple sites: the GARP study. *Annals of the Rheumatic Diseases*.

[29] Khan A. N., Jacobsen H. E., Khan J. (2017). Inflammatory biomarkers of low back pain and disc degeneration: a review. *Annals of the New York Academy of Sciences*.

[30] Goode A. P., Marshall S. W., Kraus V. B. (2012). Association between serum and urine biomarkers and lumbar spine individual radiographic features: the Johnston County Osteoarthritis Project. *Osteoarthritis and Cartilage*.

[31] Guo W., Zhang B., Li Y. (2017). Gene expression profile identifies potential biomarkers for human intervertebral disc degeneration. *Molecular Medicine Reports*.

[32] Benabdoun H. A., Kulbay M., Rondon E. P. (2019). In vitro and in vivo assessment of the proresolutive and antiresorptive actions of resolvin D1: relevance to arthritis. *Arthritis Research & Therapy*.

[33] Lee D. C., Adams C. S., Albert T. J., Shapiro I. M., Evans S. M., Koch C. J. (2007). In situ oxygen utilization in the rat intervertebral disc. *Journal of Anatomy*.

[34] Le Maitre C. L., Freemont A. J., Hoyland J. A. (2007). Accelerated cellular senescence in degenerate intervertebral discs: a possible role in the pathogenesis of intervertebral disc degeneration. *Arthritis Research & Therapy*.

[35] Yuan F. L., Xu R. S., Ye J. X., Zhao M. D., Li X., Ren L. J. (2019). Apoptotic bodies from endplate chondrocytes enhance the oxidative stress-induced mineralization by regulating PPi metabolism. *Journal of Cellular and Molecular Medicine*.

[36] Rutges J. P., Duit R. A., Kummer J. A. (2010). Hypertrophic differentiation and calcification during intervertebral disc degeneration. *Osteoarthritis and Cartilage*.

[37] Hou G., Lu H., Chen M., Yao H., Zhao H. (2014). Oxidative stress participates in age-related changes in rat lumbar intervertebral discs. *Archives of gerontology and geriatrics*.

[38] Coustry F., Posey K. L., Maerz T. (2018). Mutant cartilage oligomeric matrix protein (COMP) compromises bone integrity, joint function and the balance between adipogenesis and osteogenesis. *Matrix Biology*.

